# A prospective study examining the role of myocardial *F*ibrosis in outcome following mitral valve repair *IN DE*generative mitral *R*egurgitation: rationale and design of the mitral FINDER study

**DOI:** 10.1186/s12872-017-0715-y

**Published:** 2017-11-22

**Authors:** Boyang Liu, Nicola C. Edwards, Desley A. H. Neal, Christopher Weston, Gerard Nash, Nicolas Nikolaidis, Thomas Barker, Ramesh Patel, Moninder Bhabra, Richard P. Steeds

**Affiliations:** 1 University Hospital Birmingham NHS Foundation Trust, Birmingham, UK; 2 Institute of Cardiovascular Sciences, University of Birmingham, Birmingham, UK; 3 University Hospitals Coventry and Warwickshire NHS Trust, Coventry, UK; 4 The Royal Wolverhampton NHS Trust, Wolverhampton, UK

**Keywords:** Primary mitral regurgitation, Timing of surgery, Myocardial fibrosis, Cardiac MRI, Ventricular reverse remodelling, Extracellular volume, Arrhythmia risk, Exercise capacity

## Abstract

**Background:**

The optimal management of chronic severe primary degenerative mitral regurgitation (MR) is to repair the valve but identification of the optimal timing of surgery remains challenging. Current guidelines suggest ‘watchful waiting’ until the onset of symptoms or left ventricular (LV) dysfunction but these have been challenged as promoting ‘rescue surgery’. Better predictors are required to inform decision-making in relation to the necessity and timing of surgery. Chronic volume overload is a stimulus for adverse adaptive LV remodelling. Subclinical reduction in LV strain before mitral repair predicts a fall in LV ejection fraction following surgery and is thought to reflect the development of myocardial fibrosis in response to chronic volume overload. Myocardial fibrosis can be detected non-invasively using cardiac magnetic resonance (CMR) imaging techniques as an expansion of the extracellular volume (ECV).

**Methods/design:**

This study investigates whether: 1) patients with above median ECV will have smaller reduction in end-systolic volume index (as a measure of the degree of reverse LV remodelling) on CMR following mitral valve repair, compared to those with below median ECV; and 2) higher ECV on CMR, validated through histology, adversely impacts upon post-operative complications and symptomatic improvement following surgery.

This is a multi-centre, prospective, cross-sectional comparison of patients prior to and 9 months following surgery for chronic severe primary degenerative MR. To establish the natural history of ECV in MR, an additional cohort of patients with asymptomatic MR who do not wish to consider early repair will be followed. Investigations include CMR, cardiopulmonary exercise test, stress echocardiography, signal-averaged electrocardiogram, 24-h electrocardiogram monitoring, laboratory tests and patient-reported outcome measures. Patients undergoing surgery will have cardiac biopsies performed at the time of mitral valve repair for histological quantification of fibrosis.

**Discussion:**

This study will advance our understanding of ventricular remodelling in MR, its impact on patient symptoms and ventricular response following surgery. Establishing the link between myocardial fibrosis (measured on CMR and validated through histology), with early ventricular dysfunction, will offer physicians a novel non-invasive biomarker that can further inform the timing of surgery.

**Trial registration:**

This trial was registered at ClinicalTrials.gov (NCT02355418) on 30th November 2015.

## Background

### Challenges in determining the optimal timing of surgery

Mitral regurgitation (MR) is the most common form of valvular disease in the USA, affecting 2.5% of the general population and over 10% of those aged 75 years and older [1]. It is the second most frequent valve disease requiring surgery in Europe, and the prevalence of moderate-to-severe MR is expected to double by 2030 due to an ageing population [2]. Improvements in the diagnosis, quantification and surgical techniques for repair of a primary degenerative mitral valve (MV) now allow restoration of normal life expectancy following surgery [3].

Current AHA/ACC/ESC guidelines recommend expectant management until class 1 indications for surgery are reached, namely development of NYHA II-IV symptoms; LV ejection fraction (LVEF) <60% or LV internal diameter in systole ≥40 mm [4, 5]. Unfortunately, in spite of clear guidelines on the timing of surgery, a fifth of patients with severe primary MR continue to present post-operatively with reduced ejection fraction and an increased risk of congestive cardiac failure [6] despite single surgeon [7] and single centre [8] studies suggesting that better outcomes may be achieved. Class IIa indications for (MV) repair in asymptomatic patients at low surgical risk are also widely followed, although there is evidence for this from only one randomised study [9]. Proponents for earlier surgery make reference to registry data showing that repair in asymptomatic MR may result in better LV function, lower rates of heart failure and stroke [10], and normal life expectancy [7].

Careful surveillance with surgery delayed until class 1 indications are met, however, may deliver outcomes that are equally as good as early MV repair. In a study of 132 consecutive patients, a programme of regular (3–12 monthly) review with referral based on Class 1 indications delivered survival rates equivalent to the general population, both in those proceeding with surgery and in those who were managed conservatively; 55 ± 6% avoided surgery altogether at 8 years without compromising their outcome [11]. Since primary MR increases in prevalence with age and given the ageing of the population in Europe, there are likely to be increasing numbers of patients who may wish to avoid surgery while they feel well, providing their long-term outcome is not compromised. Moreover, there is the potential for problems with an approach that pushes asymptomatic patients through major cardiothoracic surgery in the absence of randomised trial evidence. Firstly, by definition, surgery cannot improve quality of life if the individual is truly asymptomatic but does commit the patient to a period of rehabilitation and is associated with a minimum mortality risk of 1%. Secondly, the ability to identify a reparable MV is imperfect and not everyone who expects a repair receives one. In the UK, only 64.6% of patients undergo repair but in the study by Kang et al. which recruited only those with ‘reparable’ valves, 6% still ended up with replacement [9]. Operative mortality in those planned for repair but ultimately requiring replacement is high. Finally, although MV repair is usually successful and long-lasting, this is not always the case (reoperation rate of 11–28% at 15 years) with one third having a late recurrence of moderate or severe MR [12]. In the absence of large-scale randomised data, debate continues [13, 14].

### Ventricular remodelling to guide the timing of surgery

Whilst patients may possess the same degree of MR for a similar duration, their ventricular function and symptom status may be quite different, suggesting that differential ventricular remodelling may be a potential underlying factor that influences the onset of surgical indications. In contrast to the traditional concept that there is a transition point from ‘compensated’ to ‘decompensated’ chronic primary MR, there is strong evidence that left ventricular (LV) remodelling is progressive from the earliest stages of MR with a reduction in early myocardial velocity and global strain [15, 16]. This sub-clinical reduction in contractility is in turn reflected in LA dilatation due to elevated LV end-diastolic pressure [17] and progressive secondary right ventricular failure [18]. Sub-clinical LV dysfunction is measured through change in strain via a number of methodologies, including tissue Doppler, speckle tracking echocardiography and feature-tracking CMR (FT-CMR), and predicts both the onset of symptoms [16] and the degree of reverse LV remodelling following MV repair [19]. Sub-clinical changes in LV systolic deformation, impaired diastolic relaxation and elevated LA pressure are frequently ascribed to the presence of interstitial fibrosis [20], with autopsy studies confirming that this develops in patients with MR of varying degrees of severity [21].

Characterising change in myocardial contractility in MR by fall in ejection fraction, increase in linear dimension and volume, or change in strain, whether using echocardiography or CMR, is difficult because all these techniques are volume-dependent. The evolution of CMR T1 mapping techniques offers a completely different approach that is non-invasive, quantitative but is not dependent on chamber size or volume status, since the aim is to identify interstitial myocardial fibrosis as a marker of ‘myocardial stress’ in MR [22]. T1 mapping has been validated against histology as a method for measuring interstitial fibrosis mainly in aortic stenosis, inherited cardiomyopathies and all-cause heart failure [23–25] with increasing T1 and ECV associated with increased morbidity and mortality [26, 27]. Sequences allow construction of parametric pixel-wise colour T1 map to quantitatively measure the longitudinal myocardial relaxation time before and after gadolinium contrast administration for measurement of T1 times and calculation of myocardial ECV. Using this technique, a strong linear correlation has been demonstrated between ECV and histological collagen volume fraction measured in aortic stenosis and explanted hearts of patients undergoing heart transplantation [28]. In a pilot study of 24 patients (mean age 62 +/− 16 years) with primary degenerative moderate or severe asymptomatic MR with no class I indication for surgery, we previously found a significant increase in ECV compared to age and gender-matched controls [29]. The extent of fibrosis correlated both with exercise capacity and a reduction in longitudinal strain.

Therefore, this study aims to assess whether left ventricular fibrosis as measured through CMR impacts upon patient outcomes including ventricular recovery following surgery, post-operative complications and symptomatic improvement. The primary endpoint of this study is the reduction in end-systolic volume index on CMR (as a measure of reverse LV remodelling) at 9 months following surgery, comparing those with ECV measured pre-operatively above and below the median. The reduction in left ventricular end-systolic volume index (LVESVi) is a commonly measured outcome parameter for the quantification of reverse left ventricular remodelling in MR [9, 30]. Secondary end-points (Table 2) assess the difference in exercise performance, symptom status, post-operative recovery and ventricular contractility (measured by LVEF and myocardial strain) between patients with an above and below-median ECV before and 9 months after MV surgery.

## Methods/design

### Study design

**Fig. 1:**
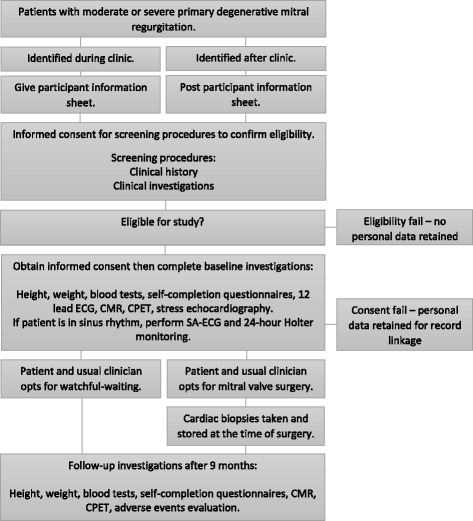
Study design flowchart

This is a multicentre prospective, cross-sectional comparison of patients with primary degenerative MR before and after MV repair. To establish the natural history of ECV in MR, an additional cohort of patients with asymptomatic MR who are not eligible or do not wish to consider early MV repair (via a class IIa surgical indication) will be followed. Study design is illustrated in Fig. 1; details of the inclusion and exclusion criteria and the study endpoints are detailed in Tables 1 and 2 respectively.

**Table 1:** Inclusion and exclusion criteria

Inclusion criteria
Age 18 and over
Primary degenerative mitral regurgitation (diagnosed and quantified according to the European Association of Echocardiography guidelines)
Exclusion criteria
Secondary mitral regurgitation
Primary mitral regurgitation not due to degenerative disease (including rheumatic disease)
Co-existing moderate or severe aortic valve disease
Congenital heart disease
Inherited or acquired cardiomyopathy
Non-incidental or symptomatic coronary artery disease
Uncontrolled atrial fibrillation (resting heart rate > 100/min)
Pregnancy
Unable to undergo CMR
Unable to undergo cardiopulmonary exercise test

**Table 2:** Primary and secondary study end points

Primary end point
Change in end-systolic volume index on CMR (as a measure of reverse LV remodelling)
Secondary end points
Exercise capacity assessed by peak VO_2_ using treadmill or bicycle
Health related quality of life assessed using the functional domain of SF-36
Extracellular volume on CMR
Ejection fraction and global myocardial strain on CMR
Measures of surgical outcome including inotrope and IABP usage, time to extubation, ITU and hospital length of stay, post-operative weight gain (pre-op to post-op day 4)

The surgical centres participating in the trial will be the University Hospital Birmingham NHS Foundation Trust, University Hospitals Coventry and Warwickshire NHS Trust, and The Royal Wolverhampton NHS Trust.

### Baseline studies

#### Cardiac magnetic resonance imaging

All patients will undergo 1.5 T multiparametric CMR at the University Hospital Birmingham site (1.5 Tesla scanner Magnetom Avanto, Siemens). Vertical long axis (VLA) and horizontal long axis (HLA) SSFP cine imaging (retrospective electrocardiographic gating, SSFP) of the left and right ventricles are performed. These images will be used to pilot the LV short axis stack acquired using serial contiguous short axis cines (typical parameters to be achieved: resolution 40–50 msec, repetition time 3.2 msec, echo time 1.7 msec, flip angle 60, field of view 300 mm, in-plane resolution 1.5 × 1.5 mm^2^, slice thickness 7 mm with 3 mm gap, minimum 25 phases per cardiac cycle) in accordance with previously validated methodology [31]. MV anatomy will be assessed using dedicated planes traversing the A1/P1 A2/P2 and A3/P3 scallops of the MV.

Analysis of ventricular function, volume and LV mass will be performed offline with delineation of papillary muscles and trabeculations using thresholding (cvi42® version 5.3.4, Circle Cardiovascular Imaging, Canada). Measurements will be made off-line using the contiguous short axis multi-slice acquisition with delineation of atria/ventricles confirmed in matched long axis planes [31]. For ventricular volume analysis, the endocardial border will be detected and the largest and smallest cavity volumes are defined as end-diastole and end-systole respectively. The endocardial border is defined as the boundary between blood pool and myocardium, with papillary muscles excluded from volumes. Mitral regurgitant volume (MRV) will be calculated as the difference between LV stroke volume (LVSV) and the aortic forward stroke volume (AoSV). Regurgitant fraction (RF) will be calculated as RF (%) = (MRV/LVSV) × 100.

Myocardial deformation will be assessed using 3D feature-tracking on CMR (FT-CMR) using cvi42® (version 5.3.4, Circle Cardiovascular Imaging, Canada). 3D FT-CMR will be performed using smoothed endocardial and epicardial borders in the end-diastolic frame of all short and long axis slices before defining the superior RV insertion points within the LV. Peak systolic strain, early diastolic strain rate and late diastolic strain rate will be recorded in the circumferential, radial and longitudinal directions.

For myocardial characterisation, myocardial and blood relaxation times pre- and 15 min after gadolinium contrast administration will be measured offline to calculate T1 times and ECV using cvi42® software as per European Society of Cardiology consensus statement [32]. All T1 maps will be checked for the presence of breathing, susceptibility and gating related artefacts. To avoid the blood-myocardial boundary, 20% offset both from the epicardial and endocardial borders will be employed and care taken to avoid any areas of LGE. Extracellular volume will be calculated using myocardial and blood T1 values before and after contrast using validated formulae [33], ECV = λ * (1 – haematocrit), where:

$$ \uplambda =\frac{\frac{\mathsf{1}}{\mathit{\mathsf{T}}{\mathsf{1}}_{\mathit{\mathsf{myo}}\;\mathit{\mathsf{post}}}}-\frac{\mathsf{1}}{\mathit{\mathsf{T}}{\mathsf{1}}_{\mathit{\mathsf{myo}}\;\mathit{\mathsf{pre}}}}}{\frac{\mathsf{1}}{\mathit{\mathsf{T}}{\mathsf{1}}_{\mathit{\mathsf{blood}\mathsf{post}}}}-\frac{\mathsf{1}}{\mathit{\mathsf{T}}{\mathsf{1}}_{\mathit{\mathsf{blood}}\ \mathit{\mathsf{pre}}}}} $$

Haematocrit for ECV calculation is be measured contemporaneously with the CMR study.

#### Cardiopulmonary exercise test

Patients will undergo cardiopulmonary exercise testing (CPET) on a treadmill using an incremental RAMP protocol based on American Thoracic Society guidelines [34]. A RAMP slope, based on gender, age, and estimated physical fitness of the subject will be set to obtain a test of approximately 10 min duration. Subjects will wear a tight facemask to permit continuous measurements of ventilation, oxygen consumption (VO_2_), and carbon dioxide production (VCO_2_) in expired gas, from which estimates of peak oxygen consumption (peak VO_2_) and anaerobic threshold (AT) can be obtained using a V-slope method [35].

#### Transthoracic echocardiography

Transthoracic echocardiography will be performed by experienced echocardiographers accredited through the British Society of Echocardiography (BSE). LV and right ventricular (RV) dimensions, atrial dimensions, systolic and diastolic function will be assessed according to the requirements for the performance of a standard transthoracic echocardiogram according to BSE guidelines [36]. Estimated pulmonary artery systolic pressure will be derived from the regurgitant jet of tricuspid regurgitation calculated by the modified Bernoulli equation and allowance made for right atrial pressure. Myocardial tissue velocity and deformation will be assessed using 2D speckle tracking [37].

#### Stress echocardiography

Stress echocardiography will be carried out at the baseline visit according to previously described methods [38], but in brief, semi-supine bicycle ergometry will be performed initially at workloads of 20-25 W for 2 min, then increased in steps of 15-30 W every 2 min as decided according to the physical fitness and symptom status of the patient. During each 2-min interval, MR quantification and estimations of LV filling pressure and pulmonary artery pressure are performed (Epic, Phillips). LV size and systolic function are assessed at baseline and peak exercise. Global longitudinal strain will be derived from the apical 2–3- and 4-chamber views via speckle tracking at baseline and peak exercise. The quantification of MR will be carried out using the integration of quantitative and qualitative approaches [39]; ‘quantitatively’ by subtracting the stroke volume across the left ventricular outflow tract (LVOT) from the stroke volume across the MV annulus, or the flow convergence method by proximal isovelocity surface area; and ‘qualitatively’ by visual assessment of the colour flow jet area and flow convergence zone.

#### Electrocardiogram

A standard 12 lead electrocardiogram (ECG) will be performed for the assessment of baseline heart rate, rhythm, and QRS morphology, followed by signal-averaged ECG (SA-ECG) for the assessment of ventricular late potentials in those with sinus rhythm. SA-ECG will be performed by averaging 250 beats using a MAC® 5500 system (GE Healthcare, Illinois, U.S.A.) at a filter setting of 40-250 Hz and aiming for a noise level of <0.3 μV.

#### 24-h Holter monitoring

3-channel 24-h Holter ECG recordings will be carried out using Lifecard CF monitors (Spacelabs Healthcare, Snoqualmie, U.S.A.). Holter data will be independently analysed at University Hospital Birmingham using Pathfinder SL software (Spacelabs Healthcare, Snoqualmie, U.S.A.). Supra-ventricular and ventricular ectopic burden, as well as the type and frequency of arrhythmic events, will be recorded. Time-domain analyses will be recorded from automated measurements of heart rate variability (HRV). Maximum, mean and minimum QT and QTc duration from automated software assisted analyses will be recorded according to previously established methodology [40].

#### Laboratory tests and biomarkers

All patients will undergo routine blood tests for full blood count, urea and electrolytes, liver function tests, C reactive protein, N-terminal pro-B natriuretic peptide, and high-sensitivity troponin T (lower limit of detection 5 ng/L, Roche) at the baseline and follow-up visits. Blood tests will be carried out at the Clinical Biochemical services at University Hospital Birmingham.

At initial visit, serum samples will also be stored for the measurement of collagen turnover markers including the amino terminal of type I and type III procollagen, circulating levels of matrix metalloproteinase-1 and tissue inhibitor of metalloproteinase-1 using commercially available ELISA assays.

#### Histology

Intraoperative myocardial biopsies (Tru-Cut biopsy needle) will be taken from the LV septum, anterior and posterior LV free wall. Samples will be fixed immediately in 10% buffered formalin, embedded in paraffin, and sections will be stained with Elastic Van Gieson (EHVG) for assessment of myocardial fibrosis. The EHVG stains fibrous tissue pink, elastic tissue purple and the muscle brown. Digital images (300dpi x 300dpi resolution, image size 1280 × 960 pixels) of the whole EHVG stained slide at 4× objective magnification (12 per biopsy) will be taken and analysed using Image J software (version 1.50i, National Institutes of Health, Bethesda, USA) by a specialist cardiac histopathologist, blinded to the imaging findings. The area of the tissue and the area of fibrosis will be determined by manually drawing around the outline of the tissue, and each area of pink/purple fibrosis using the surface pen on a surface book tablet. Measurements will be given in pixels and the percent fibrosis can be calculated.

#### Patient reported outcome symptom status

Subjects will be asked to provide data at entry and at 9 months post-surgery or post-first surveillance using a self-completion questionnaire (SF-36 and Minnesota Living with Heart Failure (MLWHF)) to eliminate any observer bias. The SF-36 has established validity in measurement of functional status in patients undergoing MV repair [41] but the MLWHF and SF-36 emotional score will also be collected for exploratory analysis to inform larger studies.

### Subject withdrawal

Patients may withdraw consent from the study at any time. If the withdrawal is initiated by a healthcare professional, full details for the reason for withdrawal is recorded on the case report forms. In all other cases, a simple statement reflecting patient preference is noted.

### Planned statistical analysis

All analyses will be carried out on an intention to treat basis. For all tests, summary statistics will be reported and 95% confidence intervals will be constructed where appropriate. A *P* value of <0.05 will be considered statistically significant without adjustment for multiple testing. Analyses will also be performed according to the following subgroups: severity of MR, degree of ventricular function as measured with above- and below-median global myocardial strain, presence or absence of subjective symptoms, and peak VO_2_ of ≥80% or <80% on CPET.

### Power calculation

Applying in-house pilot data and informed by previous CMR studies [42], we expect the standard deviation of LV end-systolic volume indexed to body surface area (ESVi) will be 12 ml/m^2^. A survival advantage has previously been shown with a 7 ml/m^2^ difference in ESVi from pre- to post-surgery in patients with asymptomatic MR [9]. This change has been selected as a clinically-significant difference for the study; hence it is powered on a comparison of the change in ESVi between two groups: those above and below median ECV. An independent samples t-test with 48 patients per group (96 total) and a within-group standard deviation of 12 ml/m^2^ yield a minimum detectable difference of 7 ml/m^2^ at 80% power, with alpha = 0.05. We therefore plan to recruit 115 subjects allowing for a 15% drop-out rate.

### Monitoring and safety assessments

Interventions in this study will be carried out according to the decision of the clinician responsible for the patient and are therefore independent of the study itself. Therefore, there is no formal data monitoring committee.

## Discussion

Physicians and patients with asymptomatic severe primary degenerative MR are faced with a significant conundrum. Delaying surgery until the onset of a class I surgical indication is associated with a possible outcome penalty for the patient [43]. Yet it is also difficult to advocate early surgery in the asymptomatic, elderly patient, when watchful-waiting offers a reasonable probability of remaining symptom-free for potentially the remainder of the patient’s life without penalty if an operation becomes necessary [11]. There is a clear unmet need for objective and quantitative markers of LV disease that can contribute to the decision regarding the optimal time for MV surgery.

The Mitral FINDER study aims to identify why patients with the same severity of MR can have such wide-range of symptoms before, and difference in ventricular response after surgery. By improving our understanding of ventricular remodelling in MR, and establishing the relationship between myocardial fibrosis and early ventricular dysfunction, this study will offer physicians a novel non-invasive biomarker that can further inform the timing of surgery. Measuring myocardial T1 relaxation offers a quantitative method of assessing ventricular fibrosis in primary MR that is not dependent on chamber volume and is not therefore subject to the limitations of ejection fraction in volume overload. This non-invasively measured imaging biomarker of fibrosis will be validated on myocardial biopsy performed at the time of MV repair. It is central to the hypothesis that patients with more myocardial fibrosis are at risk of sub-clinical myocardial dysfunction, followed by subjective exercise intolerance, that ultimately leads to overt cardiac dysfunction and irreversible myocardial damage that manifests as impaired reverse LV remodelling. We propose that in a population of patients with severe primary degenerative MR, without a class I indication for surgery, the presence of interstitial fibrosis as assessed by higher myocardial T1 / ECV values will identify patients with an increased risk of LV dysfunction after surgery. If this is shown, then there may be a role for routine use of CMR to risk stratify patients who would truly benefit from “early” surgery. It would also provide a rationale for larger clinical trials designed to examine cardiovascular morbidity and mortality end-points, as well as the use of anti-fibrotic treatments such as spironolactone to reverse or delay LV fibrosis in asymptomatic severe MR.

## Abbreviations

ACC: American College of Cardiology
AHA: American Heart Association
AoSV: aortic stroke volume
AT: anaerobic threshold
CMR: cardiac magnetic resonance
CPET: cardiopulmonary exercise test
ECG: electrocardiogram
ECV: extracellular volume
EF: ejection fraction
EHVG: Elastic Van Gieson
ESC: European Society of Cardiology
FT: feature tracking
Gd: gadolinium
Hct: haematocrit
HLA: horizontal long axis
HRV: heart rate variability
IABP: intra-aortic balloon pump
ITU: intensive therapy unit
LA: left atrium
LV: left ventricle
LVESVi: left ventricular end systolic volume index
LVOT: left ventricular outflow tract
LVSV: left ventricular stroke volume
MLWHF: Minnesota Living with Heart Failure
MR: mitral regurgitation
MRV: mitral regurgitant volume
MV: mitral valve
NYHA: New York Heart Association
RF: regurgitant fraction
SA-ECG: signal-averaged electrocardiogram
SSFP: steady-state free precession
VCO2: carbon dioxide production
VLA: vertical long axis
VO2: oxygen consumption
